# How to make it work: a framework for rapid research to inform evidence-based decision –making about the implementation of online learning during the COVID-19 pandemic

**DOI:** 10.15694/mep.2020.000154.1

**Published:** 2020-07-31

**Authors:** John Sandars, Poh Sun Goh

**Affiliations:** 1 Edge Hill University; 2 National University of Singapore

**Keywords:** COVID-19, online learning, research, policy

## Abstract

This article was migrated. The article was marked as recommended.

Evidence- based decision making about the implementation of online learning in medical education during the COVID-19 pandemic is a challenge for decision-makers since it is a time of rapid change. We present a new framework that offers a potential highly useful response to meet this challenge. Our proposed framework for rapid research of online learning during the COVID-19 pandemic recognises the challenge of understanding the complexity of the socio-technical system in which the online learning is implemented, including the behaviour change of individuals in the system and the system’s absorptive capacity. The framework provides a structured approach for rapid research to understand the complexity of the implementation of online learning during the COVID-19 pandemic. We recommend that rapid research to inform decision-making about the implementation of online learning during the COVID-19 pandemic should focus on early identification of the needs of the decision-makers and the use of high quality rapid research approaches to provide relevant and timely information about context, processes and outcomes.

## Background

There has been a major and rapid shift in the provision of medical education during the COVID-19 pandemic, from being mainly face to face to predominantly online. Although the future provision of medical education is unknown at the present time, it is likely that there will be increasing use of online learning across the continuum of medical education, from basic (undergraduate) to postgraduate to continuing ( Goh and Sandars, 2020). The extent to which online learning is implemented in the immediate future over the next 6 months, and also how this change is maintained in the more distant future after the next 6 months, will be determined by policy that is developed by a variety of decision-makers. These decision-makers range from educators responsible for smaller courses and modules to programme managers to institutional leads and directors of medical education to regulatory bodies.

In this Personal View, we will initially discuss how decision-makers make their decisions to develop evidence-based policy and how this process influences the provision of medical education. We will also highlight the complex process of implementing online learning in response to a new policy. An important challenge for medical education research is how to support decision-makers at a time of rapid change and we present a new practical framework for rapid research to inform evidence-based decision -making about the implementation of online learning during the COVID-19 pandemic. We were surprised that there appears to be scant discussion of rapid research in medical education, irrespective of the COVID-19 pandemic, and our thoughts about the new proposed framework have been informed by the wider literature in relation to rapid research for informing policy about implementation in healthcare.

## The challenge of evidence-based decision-making of policy for the implementation of online learning during the COVID-19 pandemic

A policy is a broad directive for how an action should occur ( Bell, 2010), such as the delivery of a curriculum using blended approaches (in which online learning is combined with face to face learning approaches) or the predominant use of mobile devices to access online learning. In medical education, the development of policy is a complex decision-making process that integrates an increasing desire to adopt an evidence based approach with other important external and internal drivers ( Bell, 2010). An evidence based medical education approach “attempts to find, critique and implement the highest quality research evidence that underpins the education provided to students” ( Brown and Williams, 2005). The external political and economic drivers include government targets for education and the available financial resources. The internal drivers include the range of available resources, including financial, expertise in the use of technology and technology infrastructure, and the mission of the medical education provider.

Using evidence to inform policy development has been widely researched in healthcare and three inter-related barriers to this complex process have been identified ( Bell, 2010). Decision-makers have to make a judgment based on the available evidence but often there are barriers, including uncertainty about the usefulness, quality and timeliness of this evidence. For example, systematic reviews that provide a synthesis of studies from across different contexts are very useful to decision-makers but they are dependent on the quality of the included research studies and also conducting both systematic reviews and research requires a significant amount of time to conduct. These factors limit the potential usefulness of ‘traditional’ approaches for conducting reviews and research at a time of rapid change, such as during the COVID-19 pandemic.

Developing evidence-based policy is usually an iterative process ( Bell, 2010), in which initial development leads to an implementation of a new intervention, with the findings from this implementation informing further development and implementation. The initial development of policy on online learning during the COVID-19 pandemic would have been informed by relevant guidelines on ‘best practice’ or previous research about online learning before the pandemic. However, future policy during the pandemic will need to be informed by the ever increasing research that is being produced during the pandemic and it is essential that this is useful to the decision-makers.

## Understanding the complex socio-technical system for the implementation of online learning during the COVID-19 pandemic

Evidence-based decision-making for implementation of an intervention in an organisation often has a simple belief that the actual outcome of the implementation will be the same as the intended outcome ( Bell, 2010). This lack of linear determinism is especially frequent when the policy relates to the use of technology, such as online learning ( O’Doherty et al., 2018). The extent to which a new technology is implemented in an organisation is a complex process of interactions within a socio- technical system. Understanding this process, in addition to measuring the educational outcomes, should be the focus of research so that it provides the essential information required by decision-makers. This understanding of the process can provide useful insights into why a new policy, and the implementation of a new intervention, such as online learning, has been successful, or unsuccessful, within a particular context.

### Understanding the complexity of the socio-technical system of an organisation

A socio-technical system perspective of an organisation highlights the complex and dynamic interaction between humans and technology within an organisation ( Choi, Dooley and Rungtusanatham, 2001):

• The human components include the individual and collective beliefs about the value and preferred approaches for using technology to achieve their goals, the impact of technology on their personal and working lives, and the available personal and organisational skills and resources to effectively use the technology.
• The technology components include the ease of use (the usability), the infrastructure to support the technology (such as internet connectivity) and how technology is applied and integrated to modify a task (such as a webinar using high quality pre-recorded videos and real-time online discussions to provide a substitute for a large audience lecture).

A further level of complexity in the system is the dynamic interaction between components across different levels of the system ( Klein and Kozlowski, 2000). There are numerous components in the micro (individual), meso (team or group), and macro (organization) levels of the system. A minor disruption of the complex interaction of components can rapidly produce a much wider impact on the other components, with an unexpected outcome that differs from the intended outcome. Research can identify the components and their complex interaction during the process of implementation and longer term implementation of a new online learning approach.

### Understanding the complexity of individual behavioural change in an organisation

A widely used theory to understand the behavioural change of individuals, and groups of individuals, within the complex socio-cultural system of an organisation in response to a new technology is the Technology Acceptance Model (TAM) ( Davis, 1989). The TAM conceptualises the personal intention to adopt a new technology as two inter-related factors: the perceived usefulness and the perceived ease of use. However, this model is likely to be too simplistic to unravel the complexity of change and there are alternative theories than can provide greater understanding. Researchers interested in change within complex socio-technical systems frequently apply the Behavioural Change Wheel (BCW-B) since it integrates several different theories about change ( Michie et al., 2011). This model considers three main factors (capability, motivation and opportunity) that influence behaviour:

• Capability - the ability to change a specific behavior, such as having the required knowledge and skills.
• Motivation - the personal beliefs about the advantages and disadvantages of the change (the usefulness and ease of use).
• Opportunity - the availability of time and resources in the external environment but also the cultural norms in the system.

### Understanding the complexity of absorptive capacity change in an organisation

The absorptive capacity of the complex socio-technical system of an organisation is a strong predictor of the implementation and also longer term maintenance of change ( Zou, Ertug and George, 2018). This concept can be simplified by considering the organisation as a sponge that absorbs the new intervention but also actively adapts itself to the intervention. This process can be considered as four key phases ( Camisón and Forés, 2010):

• Acquisition capacity - an organisation’s ability to find and make a decision about their intention to adopt and implement a new intervention.
• Assimilation capacity - an organisation’s ability to absorb the new intervention by analysing the requirements for effective implementation and planning the required changes to the processes in the organisation.
• Transformation capacity - an organisation’s ability to develop and refine the processes to facilitate the implementation of the new intervention.
• Application (or exploitation) capacity - an organisation’s ability to incorporate the intervention into their existing and future processes to implement and maintain the change over a longer time.

## A framework for rapid research to inform evidence-based decision –making for the implementation of online learning during the COVID-19 pandemic

Our proposed new framework for rapid research to inform evidence-based decision -making for the implementation of online learning during the COVID-19 pandemic consists of several essential inter-related components, including both the process and outcomes.

### Early engagement with decision-makers to identify needsand focus of interest

Understanding the needs of decision-makers is essential if the findings from research is to be useful for informing the decision-making process ( Patton, 2008). Clarification of the focus of interest is essential before embarking on any research. The focus of interest can be one or more processes and outcomes related to the implementation of the intervention. These processes can be considered to be the key human and technical components, the behaviour change of individuals and the absorptive capacity of the organization. The outcomes can be considered to be the educational outcomes and impact, such as described in the well known Kirkpatrick model ( Kirkpatrick and Kirkpatrick, 2006). The very simple message is that all the hard effort that research requires will be wasted if the findings are not relevant to the decision-maker.

### Provide greater information about the outcome and processes

Researchers need to adopt a realist perspective that considers that the outcomes of an intervention are the product of a process and the overall aim is to understand this process by considering in detail “what works, for whom, in what respects, to what extent, in what contexts, and how?” ( Wong et al., 2013).

This can be achieved by following a sequence of steps:

1. Use a structured template for data collection that considers the context, processes and outcomes.

### Context

Provide details of the specific context in which the implementation occurred. For example, the processes and outcomes of implementing webinars that are accessed by mobile devices are likely to be different if the students have to purchase their own device and pay for internet access compared with if the device is provided free, and with unlimited free internet access, by the university.

### Processes

For each of the key processes consider the facilitators (what worked well) and barriers (opportunities for improving implementation):

• Socio-technical system: human and technology components
• Behaviour change: capability, motivation and opportunity
• Absorptive capacity: acquisition capacity, assimilation capacity transformation capacity and application capacity

### Outcomes

It is important to consider the educational impact of the implementation ( Kirkpatrick and Kirkpatrick, 2006):

• Reaction: The extent to which students find the intervention useful, including relevance, and ease of use
• Learning: The increase in knowledge and skills experienced by the student.
• Behaviour: The extent to which students apply their learning in the working environment, such as healthcare.
• Results: The overall impact that the student’s performance has on their working environment, such as healthcare.

2. Collect a variety of data to provide a deeper understanding of the processes and outcomes associated with the implementation of the new intervention. In addition to data on student learning outcomes, data needs to be gathered from a variety of perspectives, such as students, educators and administrative staff, including help-desk support. Increasingly data can also be obtained by using analytics of the use of the online learning intervention, such as frequency and duration of access to online resources.

3. Data collection will usually require a mixed-method approach that combines quantitative and qualitative methods, with careful attention to appropriate rigour and following appropriate standards to ensure validity and reliability.

4. Discuss the findings with the participants who provided the data to check on both the accuracy and the interpretations that have been made by the researcher, with modification as necessary after the discussion.

5. Provide a summary to the decision-maker of the main findings that includes the researcher’s inferences about what works, for whom, in what respects, to what extent, in what contexts, and how.

### Use rapid research approaches

Rapid research approaches have the intention to provide relevant and responsive findings in shorter periods of time for informing decision-making. This is especially important at a time of rapid change in the online delivery of medical education during and after the COVID-19 pandemic.

There are guides for conducting rapid research (Riley et al., 2012), and also rapid literature reviews ( Khangura et al., 2012), and these guides highlight the importance of limiting the scope but to maintain high standards of conducting the data collection and analysis ( McNall and Foster-Fishman, 2007). The scope of rapid research can be limited by including purposive sampling of participants (such as high and low users), using structured templates based on context, processes and outcomes to guide data collection and analysis, and identifying common themes related to what works, for whom, in what respects, to what extent, in what contexts, and how. Limiting the scope of rapid reviews includes narrowing the number of databases, considering only previously published reviews and restrict the inclusion dates.

## Conclusion

Evidence- based decision making about the use of online learning in medical education during the COVID-19 pandemic is a challenge for decision-makers since it is a time of rapid change. Our proposed new framework for rapid research to inform evidence-based decision -making for the implementation of online learning during the COVID-19 pandemicrecognises the challenge of understanding implementation in a complex socio-technical system, including the complexity of the behavioural change of individuals and the complexity of the absorptive capacity for change in an organisation. The framework provides a structured approach for rapid research to understand this complexity and we recommend that rapid research to inform evidence-based decision -making for the implementation and maintenance of online learning during the COVID-19 pandemic should focus on early identification of the needs of the decision-makers and use high quality rapid research approaches to provide relevant and timely information about context, processes and outcomes.

## Take Home Messages

• Evidence- based decision making for the implementation of online learning during the COVID-19 pandemic is a challenge for decision-makers since it is a time of rapid change.
• Our proposed new framework for rapid research of the implementation e of online learning during the COVID-19 pandemic recognises the challenge of understanding the complexity of the socio-technical system in which the online learning is implemented.
• The framework provides a structured approach for rapid research to understand the complexity of the implementation of online learning during the COVID-19 pandemic.
• Rapid research should focus on early identification of the needs of the decision-makers and the use of high quality rapid research approaches to provide relevant and timely information about context, processes and outcomes.

## Notes On Contributors

John Sandars MB ChB (Hons), MSc, MD, MRCP, MRCGP, FAcadMEd, CertEd, FHEA is Professor of Medical Education and Director of Medical Education Innovation and Scholarship at Edge Hill University Medical School, Ormskirk, UK, and is Co-Chair of the AMEE Technology Enhanced Learning Committee. ORCiD: https://orcid.org/0000-0003-3930-387X

Poh Sun Goh, MBBS, FRCR, FAMS, MHPE, FAMEE, is an Associate Professor and Senior Consultant Radiologist at the Yong Loo Lin School of Medicine, National University of Singapore, and National University Hospital, Singapore. He is a graduate of the Maastricht MHPE program, a member of the AMEE TEL committee, and a Fellow of AMEE. ORCiD: https://orcid.org/0000-0002-1531-2053
