# Supplementary material for: How to make it work: a framework for rapid research to inform evidence-based decision –making about the implementation of online learning during the COVID-19 pandemic
Source: MedEdPublish (2016). 2020 Jul 31;9:154. [Version 1] doi: 10.15694/mep.2020.000154.1 (PMC10702682; doi:10.15694/mep.2020.000154.1)
Supplement: Peer review history

# Reviewer report: Reviewer response for version 1

**DOI:** 10.21956/mep.20115.r31022

**Published:** 2020-08-06

**Authors:** P Ravi Shankar

**Affiliations:** 1 American International Medical University

This review has been migrated. The reviewer awarded 4 stars out of 5 This is an interesting article. Adapting to change is always difficult and if the change is extremely rapid as during the COVID-19 pandemic, the difficulty is magnified. The challenge of doing research on adaptation of new methods of doing something is that the research is interdisciplinary, uses theories from management and human psychology and attempts to study human behavior which can be complex. The TAM model mentioned is useful but models are trying to simplify a complex process. The authors can expand on their proposed framework especially on how to make the research ‘rapid’ and ensure it reaches quickly to the decision makers. Another challenge to rapid dissemination of results to external individuals is the speed of the peer review process which can be slow. In a rapidly changing pandemic, the situation often changes by the time an article goes through the peer review and revision process and is published.

Reviewer Expertise:

NA

No decision status is available

# Reviewer report: Reviewer response for version 1

**DOI:** 10.21956/mep.20115.r31023

**Published:** 2020-08-03

**Authors:** Nilesh Kumar Mitra

**Affiliations:** 1 International Medical University

This review has been migrated. The reviewer awarded 4 stars out of 5 In this article, the authors have suggested framework for rapid research to inform evidence-based decision about implementation of online learning during COVID-19 pandemic situation . In background section, the authors have introduced the concept of evidence-based implementation of online learning. The authors have highlighted challenges in behavioral adjustment of the faculty to adapt to the needs of online teaching. On broad analysis of the published literature on the actual implementation of online learning in higher education institutes during COVID-19, it was found that lack of evidence-based use of structured online delivery platform and delivery of online learning with improper use of technology to meet the needs of the competency were some of the factors which made it challenging for the faculty to replace clinical face-to-face teaching overnight with online teaching (Debora et al., 2020; Luke & Diego, 2020). Some additions in the actual challenges in the evidence-based implementation of synchronous and asynchronous online learning during COVID-19 would have improved the background and helped the prospective readers to understand the challenges in maintaining the academic orientation during the COVID-19. The framework suggested is simple to understand and helps to understand the broad perspective of the areas of data collection for rapid analysis of the implementation of online learning. The learnability approach of the online platform and courses implemented gives a crucial insight to the educators about future interventions and gaps in the clinical competencies of the students who went through the online course. As suggested by the authors it should be a mixed-method approach, encompassing the online platform (synchronous and asynchronous with analysis of feedback), delivery, achievement of competency by the learners, as well as retention of their learning with impact on the healthcare system.

Reviewer Expertise:

NA

No decision status is available
